# *CRPPA* exon 6–9 deletion as a founder mutation in Chinese patients with dystroglycanopathy

**DOI:** 10.1002/ped4.70029

**Published:** 2025-11-30

**Authors:** Jihang Luo, Yidan Liu, Danyu Song, Shiqi Yang, Xiaona Fu, Lin Ge, Cuijie Wei, Liya Cui, Yanbin Fan, Huaxia Luo, Yanwei He, Jin Xu, Qiang Shen, Yuxuan Guo, Motoi Kanagawa, Tatsushi Toda, Jingmin Wang, Hong Zhang, Hui Xiong

**Affiliations:** ^1^ Children's Medical Center Peking University First Hospital Beijing China; ^2^ Department of Neurology Beijing Children's Hospital, Capital Medical University, National Center for Children's Health Beijing China; ^3^ State Key Laboratory of Vascular Homeostasis and Remodeling The Institute of Cardiovascular Sciences School of Basic Medical Sciences Peking University Health Science Center Beijing China; ^4^ Laboratory of Electron Microscopy Peking University First Hospital Beijing China; ^5^ Department of Cell Biology and Molecular Medicine Ehime University Graduate School of Medicine Toon Japan; ^6^ Department of Neurology Graduate School of Medicine The University of Tokyo Tokyo Japan

**Keywords:** Chinese, *CRPPA*, Dystroglycanopathies, Founder mutation, Genotype–phenotype correlation

## Abstract

**Importance:**

Dystroglycanopathies (DGPs) are a group of muscular dystrophies with abnormal glycosylation of dystroglycan. *CRPPA* is a gene associated with DGPs. Understanding the genetic basis, genotype–phenotype correlations, and population‐specific mutations is crucial for accurate diagnosis and genetic counseling.

**Objective:**

To investigate *CRPPA* mutations in Chinese pediatric patients with DGPs, analyze genotype–phenotype correlations, and determine whether specific deletions represent founder mutations in this population.

**Methods:**

Clinical and genetic data of pediatric patients with *CRPPA*‐related DGPs between June 2006 and December 2023 from Peking University First Hospital were collected and analyzed. Muscle biopsy specimens from four patients were examined using immunohistochemistry, immunofluorescence, and electron microscopy. Haplotype analysis was performed to investigate the potential founder mutation.

**Results:**

Among the 16 patients studied, phenotypes ranged from severe muscle‐eye‐brain disease to milder limb‐girdle muscular dystrophy. Twenty‐one pathogenic variants were identified, including five novel variants. A recurrent exon 6–9 deletion emerged as the second most frequent variant (25.0%, 4/16), with haplotype analysis supporting a founder mutation in Chinese patients. At follow‐up, most patients remained non‐ambulatory, and one patient died of respiratory failure.

**Interpretation:**

This study broadens the *CRPPA* mutational spectrum and identifies a founder mutation of exon 6–9 deletion in Chinese patients. These findings have important implications for population‐specific screening, diagnosis, and genetic counseling.

## Graphical abstract

Analysis of sixteen Chinese dystroglycanopathy patients reveals a founder mutation (*CRPPA* exon 6–9 deletion) in 25% of cases and expands the phenotypic spectrum from severe muscle‐eye‐brain disease to limb‐girdle muscular dystrophy.

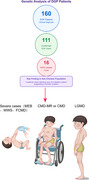

## INTRODUCTION

Dystroglycanopathies (DGPs) are a group of rare autosomal recessive muscular dystrophies characterized by defects in α‐dystroglycan (α‐DG) glycosylation, resulting in a wide clinical spectrum.^1^ These disorders are typically classified into three categories based on disease severity.^2^ The most severe phenotypes include Walker‐Warburg Syndrome, muscle‐eye‐brain disease (MEB), and Fukuyama congenital muscular dystrophy (FCMD), which typically manifest in infancy or early childhood, presenting with hypotonia and muscle weakness from birth, accompanied by ocular involvement and severe structural brain abnormalities.^3^ The intermediate type manifests as congenital muscular dystrophy (CMD) with or without mental retardation (MR), and the mildest form presents as limb‐girdle muscular dystrophy (LGMD). In clinical practice, some patients may exhibit overlapping features between CMD and LGMD, characterized by congenital onset with preserved ambulation and variable cognitive involvement, representing a phenotypic continuum between these classifications.^4^, ^5^, ^6^

To date, mutations in 19 distinct genes have been identified as causative agents of DGPs.^2^, ^7^ *CRPPA*, located on chromosome 7p21.2, encodes cytidine diphosphate (CDP)‐L‐ribitol pyrophosphorylase A, also known as isoprenoid synthase domain‐containing protein (ISPD). ISPD plays a crucial role in the biosynthesis of ribitol‐phosphate, which serves as a key building block for the glycosylation of α‐DG. This glycosylation is essential for the ability of α‐DG to bind extracellular matrix proteins, such as laminin, thereby maintaining the structural integrity of the dystrophin‐glycoprotein complex.^8^, ^9^ It has 10 exons and encodes a 451 amino acid protein with an N‐terminal catalytic domain (amino acids 47–273) and a C‐terminal non‐catalytic domain (amino acids 283–451).^10^ A broad spectrum of *CRPPA* variants, including missense, nonsense, splice‐site, and frameshift mutations, has been reported worldwide.^11^, ^12^, ^13^, ^14^, ^15^ However, systematic genotype–phenotype correlations and population‐specific genetic features, especially in the Chinese population, remain poorly defined, limiting diagnostic precision and genetic counseling.

In this study, we retrospectively analyzed a large cohort of Chinese pediatric patients with clinically suspected DGPs. Through genomic analysis, 16 patients harboring pathogenic *CRPPA* variants were identified, and detailed clinical, genetic, and pathological characterizations were performed. Importantly, an identical exon 6–9 deletion with consistent breakpoints was discovered to be carried by four unrelated patients, and a founder mutation in the Chinese population was suggested by haplotype analysis. Our findings expand the mutational spectrum of *CRPPA* and provide novel insights into population‐specific genetic features with direct implications for diagnostic strategies and genetic counseling.

## METHODS

### Ethical approval

This study was approved by the Ethics Committee of the Peking University First Hospital (No. 2015[916], Beijing, China). Written informed consent to participate in this study was obtained from the participants’ legal guardians or their next of kin.

### Patient registration and clinical data collection

Between June 2006 and December 2023, 160 patients with a clinical diagnosis of DGPs were evaluated at the Peking University First Hospital. All patients underwent genomic testing, including targeted myopathy gene panel sequencing, whole‐exome sequencing (WES), or whole‐genome sequencing (WGS). Clinical information, including demographic data, clinical manifestations, laboratory findings, neuroimaging, and follow‐up records, was retrospectively collected from the medical charts.

The diagnosis of *CRPPA*‐related DGPs was established when pathogenic or likely pathogenic variants in the *CRPPA* gene were identified through genetic testing in accordance with the American College of Medical Genetics and Genomics (ACMG) guidelines. Patients with confirmed *CRPPA* variants were included in the present study for detailed clinical and genetic characterization.

### Genetic analysis

Genomic DNA was extracted from the peripheral blood samples. Sequencing and variant detection were performed at Beijing Chigene Translational Medicine Research Center. Minor allele frequencies (MAFs) were annotated using multiple population databases, including 1000 Genomes, dbSNP, ESP, and ExAC, and an in‐house database.

Variant pathogenicity was evaluated according to ACMG guidelines, with reference to the OMIM, HGMD, and ClinVar databases. In silico prediction tools (Provean, SIFT, PolyPhen‐2 HDIV/HVAR, MutationTaster, M‐CAP, and REVEL) were used to assess the functional impact of protein variants. Splicing variants were analyzed using MaxEntScan, dbscSNV, and GTAG.

### Haplotype analysis

Haplotype analysis was performed in four patients (patient [P] 5, 6, 12, and 16) carrying the *CRPPA* exon 6–9 deletion to investigate the potential founder effect. Eight informative single‐nucleotide polymorphism (SNP) markers spanning the *CRPPA* gene region were selected by analyzing WGS data from these patients. The selection process consists of several steps. Variants within a 200 kb region flanking *CRPPA* were initially identified in all four patients. These variants were filtered for population frequencies greater than 0.05 in East Asian populations. The shared variants among all the patients were subsequently determined. Markers were strategically distributed to ensure adequate coverage of the gene region. The final eight markers (rs1373698562, rs1357073749, rs765283, rs6947755, rs34460874, rs6965612, rs868837227, and rs13245556) were distributed across the gene, with five markers located in the 5' region (spanning introns 4–9) and three markers in the 3' region (within intron 2). This distribution enabled comprehensive haplotype reconstruction, while maintaining sufficient spacing to distinguish between shared ancestry and independent mutational events.

### Immunohistochemistry, immunofluorescence staining, and electron microscopy

Muscle biopsy tissue was collected from P2, P6, P10, P15, alongside control individuals without DGPs. Informed consent was obtained from all participants prior to the procedure. For P2, P6, and P10, muscle tissue samples were preserved by freezing in liquid nitrogen‐cooled isopentane. Cryostat sections measuring 8 µm in thickness were prepared from frozen specimens. Sequential sections were stained with hematoxylin and eosin. For immunohistochemical analysis, we employed an α‐DG mouse monoclonal antibody (clone IIH6C4, dilution 1:20, catalog no. 05‐593; Merck Millipore, Darmstadt, Germany) along with a laminin α2 mouse monoclonal antibody (clone 5H2, dilution 1:20, catalog no. MAB1922; Merck Millipore). Sections from P6 were subjected to immunofluorescence staining using the monoclonal antibody IIH6 containing α‐DG glycan (clone IIH6C4, dilution 1:200, catalog no. 05‐593; Merck Millipore, Darmstadt, Germany) and the secondary antibody goat anti‐mouse IgG Alexa Fluor 488 (1:200 dilution; Merck Millipore, Darmstadt, Germany). Immunofluorescence images were captured using a Polaris Multispectral Tissue Imaging System (Vectra Polaris, USA). For P15, the muscle tissues were immersed in a 3% glutaraldehyde fixative solution. Ultrathin sections were prepared at the Electron Microscopy Laboratory of Peking University First Hospital. The prepared sections were subsequently observed under a Tecnai G2 20 (FEI) microscope operating at 80 kV.

## RESULTS

### Clinical features in 16 patients with *CRPPA*‐related DGPs

Between June 2006 and December 2023, 114 patients with genetically confirmed DGPs were identified in our cohort (Figure S1). Among them, *FKRP* was the most common pathogenic gene, accounting for 34.2% of cases (39 patients), followed by *POMGNT1* in 18.4% (21 patients), and *CRPPA* in 14.0% (16 patients). The remaining cases were attributed to *POMT1* (12.3%, 14 patients), *B3GALNT2* (11.4%, 13 patients), *FKTN* (5.3%, 6 patients), *POMT2* (2.6%, three patients), less frequently *GMPPB* (1.8%, two patients), and *POMGNT2* (0.9%, one patient). Among these, 16 patients (10 previously reported^2^) were diagnosed with *CRPPA*‐related DGPs, comprising ten females and six males with ages at evaluation ranging from 9 months to 15 years. Clinical phenotypes included MEB (*n* = 8), CMD (*n* = 5), LGMD (*n* = 2), and CMD/LGMD (*n* = 1) (Table 1).

**TABLE 1:** Clinical manifestations of 16 patients with *CRPPA*‐related DGPs

Patient	Phenotype	Age at evaluation/ Sex	Maximum athletic ability/age	Current athletic ability	Mental retardation	Convulsion	Eye involvement	CK (U/L)	MRI	Electrocardiogram/ Cardiac ultrasound
P1	MEB	9y5m/F	Sit/1y	Sit	Y	N	Strabismus and optic nerve atrophy of the left eye	9418	Cerebellar and brainstem dysplasia, cerebellar microcysts	Normal/Normal
P2	MEB	4y10m/M	Sit/3y	Sit	Y	Y	Strabismus, bilateral optic nerve atrophy	5003–5403	Posterior head hypothalamic gyrus malformation, cerebellar and brainstem hypoplasia, cerebellar microcystic, widened posterior horn of ventricle, occipital meningeal bulge	‐/Atrial septal defect
P3	MEB	10y8m/ F	Sit/1y7m	Sit	Y	N	Strabismus and optic nerve atrophy in the left eye	6798–14 295	Cerebellar and brainstem hypoplasia	Normal/Normal
P4	MEB	9m/M	Raise head/4m	Raise head	Y	Y	Strabismus and optic nerve atrophy of the left eye	5306–9302	Oligocerebral gyrus malformation, cerebellar microcyst, cerebellar hypoplasia	Normal/Normal
P5	MEB	3y2m/F	Help station/5y4m	Sit	Y	N	Strabismus, retinitis pigmentosa	6633–12 120	Cerebellar anomalies, cerebellar cysts	Normal/Normal
P6	MEB	15y6m/F	Sit/1y6m	Sit	Y	N	Retinal choroidal defect in the right eye with internal strabismus	2303–6736	Bilateral frontal cortical thickening and undersmoothing, brainstem cerebellar hypoplasia	‐/‐
P7	MEB	10y7m/M	Sit/2y	Sit	Y	N	Strabismus, retinitis pigmentosa	3014–8976	Cerebellar and brainstem dysplasia	‐/‐
P8	MEB	6y11m/M	Sit/3y1m	Sit	Y	N	Strabismus, bilateral optic nerve atrophy	2583–11 236	Cerebellar and brainstem dysplasia, cerebellar microcysts	‐/‐
P9	CMD without MR	12y1m dead/F	Sit/1y7m	/	N	N	N	1694–28 850	Cerebellar hypoplasia	Sinus block/‐
P10	CMD‐MR	6y3m/F	Walk/2y1m	Walk	Y	Y	N	4248–8760	Cerebellar and brainstem dysplasia, microcystic cerebellum	Normal/Patent foramen ovale
P11	CMD without MR	4y2m/F	Creep/2y	Creep	N	N	N	3402	Cerebellar hypoplasia	Normal/‐
P12	CMD‐MR	4y10m/F	Help station /2y9m	Help station	Y	N	N	500–7000	Oligocerebellar gyrus malformation, microcystic cerebellum, cerebellar hypoplasia	Normal/Normal
P13	CMD‐MR	6y/M	Help station/5y4m	Help station	Y	N	N	4399–10 303	Oligocerebellar gyrus malformation, microcystic cerebellum, cerebellar and brainstem dysplasia	Normal/Normal
P14	CMD/LGMD	6y1m/M	Walk/1y6m	Walking	N	N	N	357–11 035	Bilateral thickening and undersmoothing of the frontal cortex, brainstem cerebellar hypoplasia	Sinus arrhythmia/Tricuspid regurgitation
P15	LGMD	13y5m/F	Walk/1y2m	Walk	N	N	N	9000–16 264	Cerebellar and brainstem hypoplasia	Normal/Normal
P16	LGMD	12y6m/F	Walk/1y2m	Walk	N	N	N	1797–7116	Cerebellar and brainstem hypoplasia, cerebellar microcapsules	‐/Left ventricular false tendon

Abbreviations: CK, creatine kinase; CMD, congenital muscular dystrophy; *CRPPA*, CDP‐L‐ribitol pyrophosphorylase A; DGPs, dystroglycanopathies; F, female; LGMD, limb‐girdle muscular dystrophy; M, male; m, month; MEB, muscle‐eye‐brain disease; MR, mental retardation; MRI, magnetic resonance imaging; N, no such symptom; Y, have the symptom; y, year.

The MEB subgroup (P1–P8) represented the most severe phenotype, with universal ocular involvement, such as strabismus and optic nerve atrophy, and seven of eight presented with intellectual disability. None of the patients achieved independent ambulation, indicating profound motor impairment. The CMD subgroup (P9–P13) displayed a relatively milder phenotype; four of the five patients had intellectual disability, while one (P10) attained independent walking. A single CMD/LGMD patient (P14) exhibited a distinctive profile, retaining walking ability despite marked brain abnormalities and cardiac involvement. The LGMD subgroup (P15–P16) demonstrated the mildest presentation with preserved cognition and independent walking.

Across all subgroups, the patients exhibited varying degrees of hypotonia and delayed motor milestones, with the most profound impairment observed in the MEB subgroup. Several patients with MEB never achieved basic motor skills, and some were unable to sit independently. Three patients (P2, P4, and P10) experienced epileptic seizures, including two with MEB and one with CMD. Ocular involvement was a consistent feature in all MEB cases, whereas patients with CMD and LGMD showed no ocular abnormalities.

**FIGURE 1:**
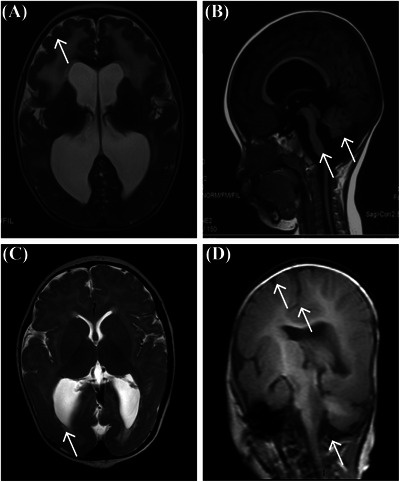
Cerebellar and brainstem abnormalities on brain MRI in patients with *CRPPA*‐related dystroglycanopathy. Representative images from four patients show consistent patterns of cerebellar malformation. (A) Patient 2, anteriorly enlarged cerebellum; (B) Patient 10, cerebellar tonsillar malformation and brainstem dysplasia; (C) Patient 13, cerebellar cyst; (D) Patient 11, reduced cerebellar hemispheres and brainstem dysplasia. White arrows indicate the representative abnormalities. *CRPPA*, CDP‐L‐ribitol pyrophosphorylase A; MRI, magnetic resonance imaging.

All patients exhibited elevated creatine kinase levels ranging from 500 U/L to 28 850 U/L (reference: 25–200 U/L). Brain magnetic resonance imaging consistently revealed abnormalities across all phenotypic groups, most commonly cerebellar and brainstem dysplasia, irrespective of mutation type. The severity of structural brain abnormalities correlated closely with the disease phenotype, with MEB patients presenting the most extensive malformations, such as cerebellar microcysts and posterior horn dilatation, whereas CMD patients displayed milder cerebellar and brainstem hypoplasia (Figure 1).

**FIGURE 2:**
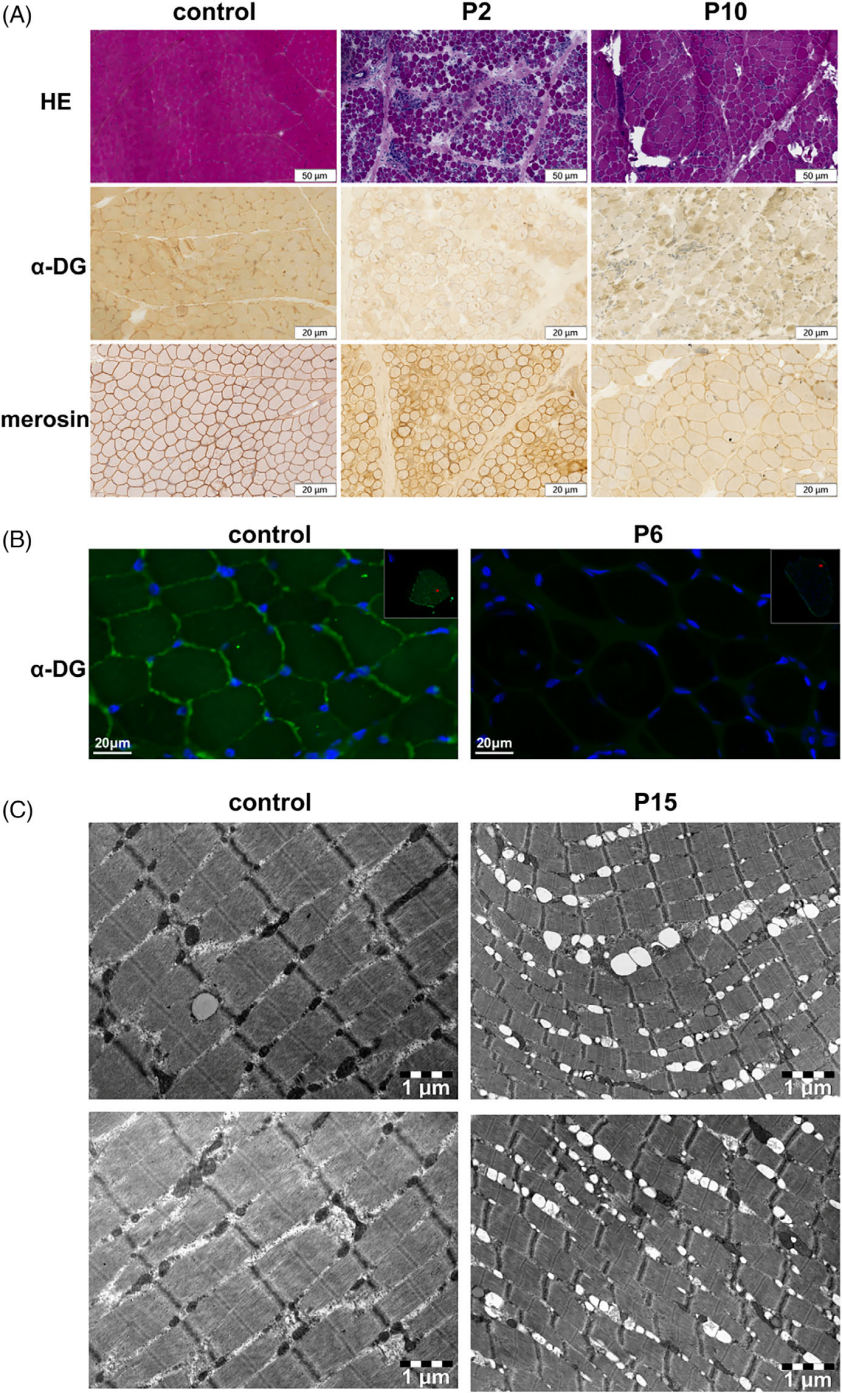
Skeletal muscle biopsy pathological analysis. (A) Hematoxylin‐eosin (HE) staining of patient samples (P2 and P10) demonstrated similar pathological findings, including increased variation in muscle fiber diameter with coexistence of atrophic and hypertrophic fibers, proliferation of endomysial connective tissue, rounded muscle fiber morphology, and muscle fiber necrosis, regeneration, and nuclear internalization. Immunolabeling for α‐dystroglycan (α‐DG) with IIH6 antibody in patient samples showed reduced glycosylated α‐DG staining compared to control. The expression of laminin α2 (merosin) was essentially normal across all samples. Scale bars: 50 µm for HE staining, 20 µm for immunohistochemistry staining. (B) Muscle immunofluorescence staining suggested a marked deficiency in glycosylation of α‐DG in Patient 6 (P6) (Blue indicates DAPI, green indicates α‐DG IIH6). (C) Electron microscopy results of muscle tissue in Patient 15 (P15). Morphological abnormalities of mitochondria were observed, with heterogeneity in size compared to normal mitochondria. Swelling and volume enlargement of mitochondria were detected, and the distribution between muscle fibers was found to be disorganized. Additionally, the normal cristae structure within the mitochondria was no longer visible. Participants without dystroglycanopathy served as controls.

Typical dystrophic changes were revealed by histopathological examination of the muscle biopsy specimens from P2 and P10. Similar findings were demonstrated by hematoxylin and eosin (HE) staining in both patients, including increased variation in muscle fiber diameter, proliferation of fatty connective tissue within the endomysium, muscle fiber regeneration, necrosis, and nuclear internalization. The key finding was that significantly reduced or absent α‐DG expression was observed on immunostaining using the IIH6 antibody in both P2 and P10, indicating defective α‐DG glycosylation consistent with α‐dystroglycanopathy (Figure 2A). Immunofluorescence analysis with the primary antibody α‐DG glycan IIH6 demonstrated a marked deficiency in α‐DG glycosylation in P6 (Figure 2B). Electron microscopy of the muscle tissue from P15 (LGMD type) further revealed pronounced mitochondrial abnormalities. The mitochondria exhibited morphological heterogeneity with irregular sizes compared with normal controls. Prominent swelling and enlargement were observed, accompanied by a disorganized distribution of the muscle fibers. Moreover, the normal cristae structure within the mitochondria was completely disrupted and no longer visible (Figure 2C).

### Genetic analysis

**FIGURE 3:**
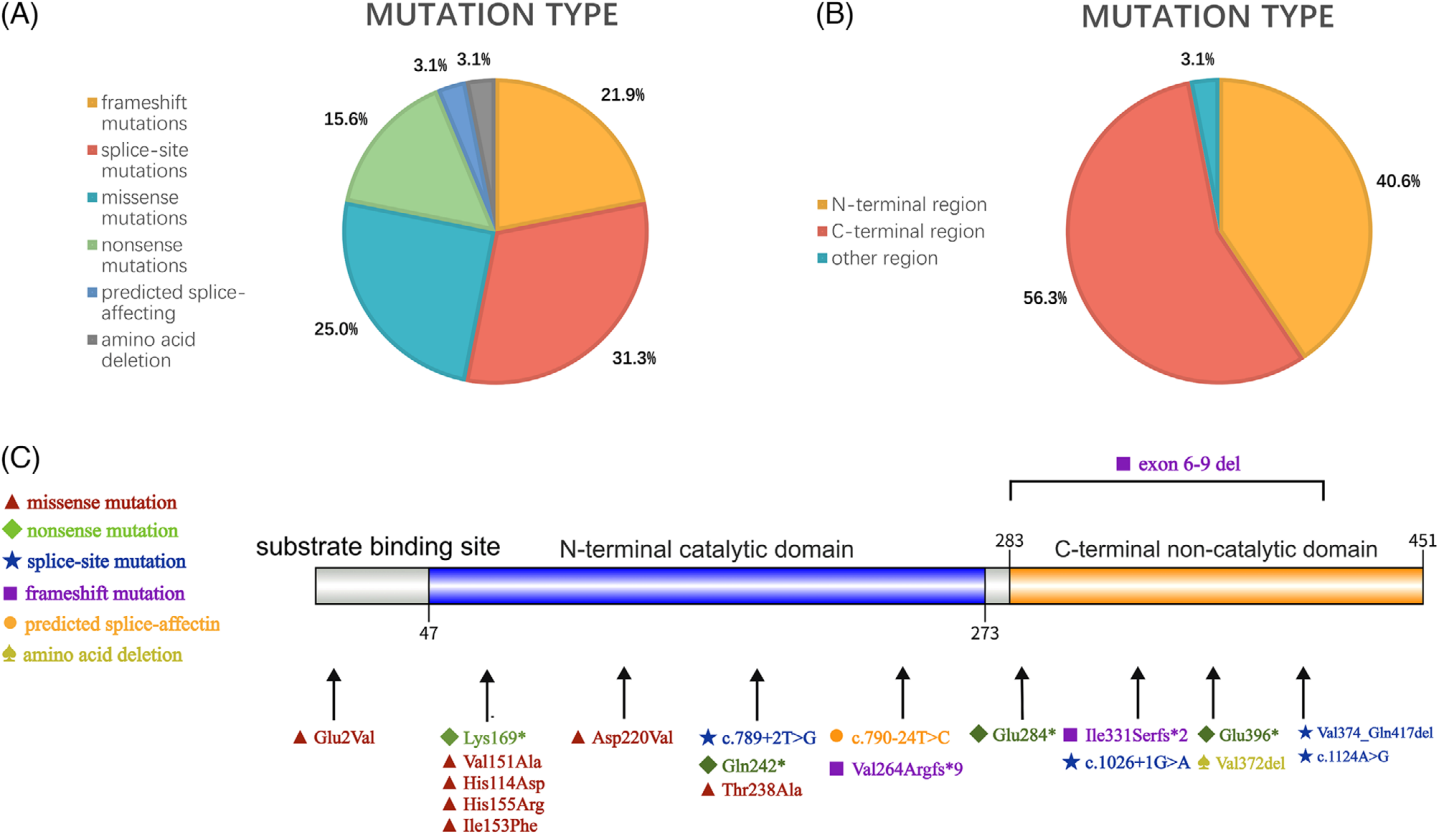
Distribution of mutation types in *CRPPA*‐related DGPs. (A) Among the 32 pathogenic variants identified in 16 patients with *CRPPA*‐related DGPs, splice‐site mutations were the most common (31.3%), followed by missense mutations (25.0%), frameshift mutations (21.9%), and nonsense mutations (15.6%). Additionally, predicted splice‐affecting mutations and amino acid deletions each accounted for 3.1% of the variants. (B) Mutation distribution by protein domain in *CRPPA* showed that the majority of mutations were located in the C‐terminal region (56.3%), followed by the N‐terminal region (40.6%), with a small proportion (3.1%) found in other regions. (C) Mutation mapping of the *CRPPA* gene. The schematic representation shows the *CRPPA* protein containing a substrate binding site, an N‐terminal catalytic domain (blue), and a C‐terminal non‐catalytic domain (orange). Specific mutations are mapped to their respective locations using colored symbols. *CRPPA*, CDP‐L‐ribitol pyrophosphorylase A; DGPs, dystroglycanopathies.

A total of 21 distinct variants, including five novel and 16 previously reported pathogenic variants, were identified in 32 alleles from 16 patients with *CRPPA*‐related DGPs (Table 2). Compound heterozygous mutations were present in the majority of patients (15/16, 93.8%), while a homozygous mutation was identified in one patient (P9). Among the 32 alleles, the variants included splice‐site mutations (10/32, 31.3%), missense mutations (8/32, 25.0%), frameshift mutations (7/32, 21.9%), nonsense mutations (5/32, 15.6%), predicted splice‐affecting mutation (1/32, 3.1%), and amino acid deletion (1/32, 3.1%) (Figure 3A).

**TABLE 2:** Genotype of the patients with *CRPPA*‐related DGPs

Patient	Exon	Nucleotide change	Predicted amino acid changes	Type of mutation	Source of mutation	Novel/ Reported	N‐terminus or C‐terminus
P1	1	c.5A>T	p.Glu2Val	missense	Maternal	Reported	None
	2	c.505A>T	p.Lys169*	nonsense	Paternal	Reported	N
P2	4	c.789+2T>G	/	splice‐site	Paternal	Reported	N
	9	c.1251G>A	p.Val374_Gln417del	splice‐site	Maternal	Reported	C
P3	7	c.990delC	p.Ile331Serfs*2	frameshift	Maternal	Reported	C
	9	c.1251G>A	p.Val374_Gln417del	splice‐site	Paternal	Reported	C
P4	4	c.724C>T	p.Gln242*	nonsense	Paternal	Novel	N
	9	c.1251G>A	p.Val374_Gln417del	splice‐site	Maternal	Reported	C
P5	2	c.452T>C	p.Val151Ala	missense	Paternal	Novel	N
	6–9	exon 6–9 del	/	frameshift	Maternal	Reported	C
P6	5	c.790‐24T>C	/	predicted splice‐affecting	Paternal	Novel	N
	6–9	exon 6–9 del	/	frameshift	Maternal	Reported	C
P7	5	c.790‐12T>C	p.Val264Argfs*9	frameshift	Maternal	Novel	N
	6	c.850G>T	p.Glu284*	nonsense	Paternal	Novel	C
P8	5	c.790‐12T>C	p.Val264Argfs*9	frameshift	Maternal	Novel	N
	6	c.850G>T	p.Glu284*	nonsense	Paternal	Novel	C
P9	2	c.340C>G	p.His114Asp	missense	Paternal	Reported	N
	2	c.340C>G	p.His114Asp	missense	Maternal	Reported	N
P10	2	c.464A>G	p.His155Arg	missense	Paternal	Reported	N
	4	c.712A>G	p.Thr238Ala	missense	Maternal	Reported	N
P11	3	c.659A>T	p.Asp220Val	missense	Maternal	Reported	N
	9	c.1251G>A	p.Val374_Gln417del	splice‐site	Paternal	Reported	C
P12	6–9	exon 6–9 del	/	frameshift	Maternal	Reported	C
	9	c.1251G>A	p.Val374_Gln417del	splice‐site	Paternal	Reported	C
P13	8	c.1186G>T	p.Glu396*	nonsense	Paternal	Reported	C
	9	c.1251G>A	p.Val374_Gln417del	splice‐site	Maternal	Reported	C
P14	7	c.1026+1G>A	/	splice‐site	Paternal	Reported	C
	8	c.1114_1116delGTT	p.Val372del	amino acid deletion	Maternal	Reported	C
P15	7	c.1026+1G>A	/	splice‐site	Paternal	Reported	C
	9	c.1124A>G	/	splice‐site	Maternal	Reported	C
P16	2	c.457A>T	p.Ile153Phe	missense	Maternal	Reported	N
	6–9	exon 6–9 del	/	frameshift	Paternal	Reported	C

Genetic data for 10 of the 16 patients have been published.^2^

None, not at either the N‐terminus or C‐terminus. *CRPPA*, CDP‐L‐ribitol pyrophosphorylase A; DGPs, dystroglycanopathies.

The variant c.1251G>A (p.Val374_Gln417del) was identified in six patients (P2, P3, P4, P11, P12, and P13), representing the most frequent pathogenic variant in the cohort (6/16, 37.5%). The exon 6–9 deletion was identified in four unrelated patients (P5, P6, P12, and P16), making it the second most common variant in the cohort (4/16, 25.0%). This large deletion leads to an out‐of‐frame mutation, resulting in premature termination of translation. In total, 18 of the 32 variants were located in the non‐catalytic C‐terminal domain (amino acids 283–451) of the CRPPA protein, exceeding the 13 variants in the N‐terminal catalytic domain (amino acids 47–273) (Figure 3B, C).

The ACMG classification of mutations in the 15 patients indicated that some mutations were pathogenic or likely pathogenic. However, one splice‐affecting variant, c.790‐24T>C, in P6 was predicted to have uncertain pathogenicity. Notably, immunofluorescence staining revealed deficient α‐DG glycosylation in this patient, confirming the functional impact of this variant (Figure 2B).

### Haplotype analysis and founder mutation in four patients

**FIGURE 4:**
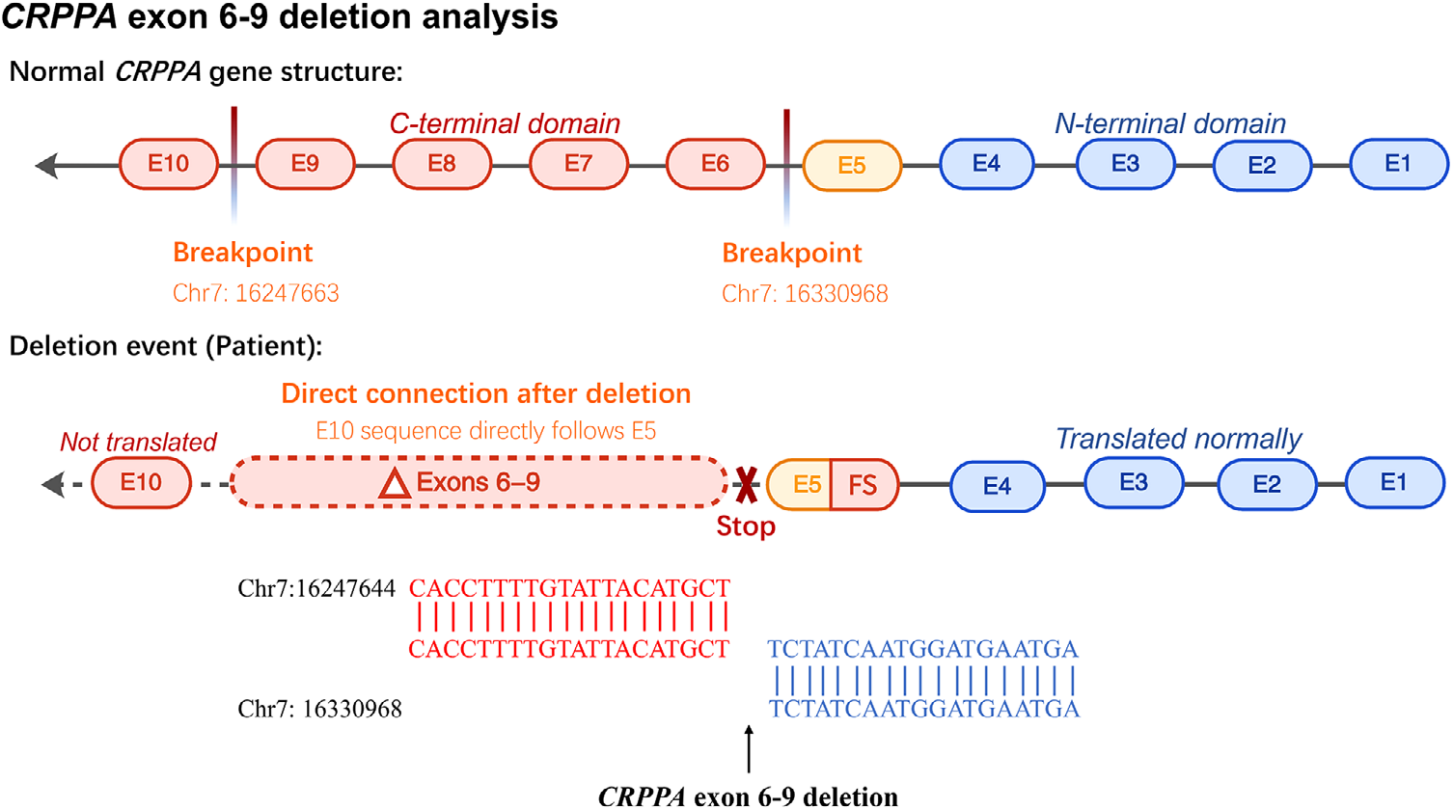
Structural diagram of *CRPPA* gene exon 6–9 deletion analysis. The normal *CRPPA* gene structure shows 10 exons (E1–E10) with the C‐terminal domain (E6–E10, red) and N‐terminal domain (E1–E5, blue). In the deletion event observed in patients, exons 6–9 are deleted, creating a direct connection between E10 and E5 sequences. This results in a frameshift mutation with premature stop codon formation. All 4 patients showed identical deletion breakpoints on chromosome 7: upstream breakpoint at chr7: 16247663 (red sequence: CACCTTTTGTATTACATGCT) and downstream breakpoint at chr7:16330968 (blue sequence: TCTATCAATGGATGAATGA). The remaining exons E1–E5 are translated normally, while E10 is not translated due to the frameshift. *CRPPA*, CDP‐L‐ribitol pyrophosphorylase A.

Among the C‐terminal variants, four non‐consanguineous patients harbored a recurrent deletion of exons 6–9. These four patients shared identical breakpoints (GRCh37.p13 [GCF_000001405.25]) and the same deletion size spanning chr7:16247663 and chr7:16330968 (Figure 4).

To investigate whether this recurrent deletion originated from a common ancestral allele, haplotype analysis was performed on P5, P6, P12, and P16, all of which were heterozygous for this deletion. Eight SNP markers around the *CRPPA* gene region were analyzed (Table 3). The analyzed SNPs included rs1373698562, rs1357073749, rs765283, rs6947755, rs34460874, rs6965612, rs868837227, and rs13245556. Remarkably, all four patients shared an identical haplotype across these SNP markers with the allele pattern A‐G‐G‐G‐insT‐G‐A‐A. This specific haplotype was absent in randomly selected, ethnically matched control samples, strongly indicating that the exon 6–9 deletion represents a founder mutation in the Chinese population (Table 3).

**TABLE 3:** Results of the haplotype analysis

Variable	rs1373698562	rs1357073749	rs765283	rs6947755	rs34460874	rs6965612	rs868837227	rs13245556
SNP of *CRPPA* (NM_001101426)	c.1251+49664G>A	c.1251+34470A>G	c.1251+24826C>G	c.1251+16638C>G	c.789+607_c.789+608insT	c.535‐10845A>G	c.535‐13685T>A	c.535‐13687T>A
Patient 5	A	G	G	G	insT	G	A	A
Patient 6	A	G	G	G	insT	G	A	A
Patient 12	A	G	G	G	insT	G	A	A
Patient 16	A	G	G	G	insT	G	A	A

Abbreviation: *CRPPA*, CDP‐L‐ribitol pyrophosphorylase A; SNP, single‐nucleotide polymorphism.

### Genotype–phenotype correlations

Frameshift mutations in compound heterozygous combinations were predominantly associated with severe phenotypes. All patients carrying the exon 6–9 deletion showed variable phenotypes; among them, three patients (P5, P6, and P12) presented with MEB or CMD‐MR, while one patient (P16) was diagnosed with LGMD, suggesting that even identical mutations may result in variable clinical outcomes when combined with different second mutations. Other patients carrying dual protein‐truncating mutations (P7 and P8) both presented with MEB, indicating a tendency toward severe phenotypes for frameshift mutations.

Splice site mutations showed a broader phenotypic range. The recurrent variant c.1251G>A (p.Val374_Gln417del) was identified in six patients with varying severities: three patients (P2, P3, and P4) presented with MEB, two patients (P12, P13) had CMD‐MR, and one patient (P11) had CMD without MR. This variability indicated that splice‐site mutations could be associated with severe, intermediate, and milder phenotypes, depending on the second mutation in the compound heterozygous state.

Missense mutations are associated with variable phenotypes across the spectrum. These mutations were found in patients with MEB (P1, P5), CMD without MR (P9, P11), CMD‐MR (P10), and LGMD (P16), showing no clear genotype–phenotype correlation for this mutation type in compound heterozygous combinations. Notably, P9 carried a homozygous missense mutation (p.His114Asp), but presented only with CMD without MR, suggesting that certain missense mutations may retain partial protein function.

In summary, frameshift mutations were associated with severe disease phenotypes, whereas splice‐site and missense mutations showed significant phenotypic heterogeneity. In compound heterozygous patients, the final phenotypic severity depends on the cumulative effect of both mutations rather than any single mutation type.

### Follow‐up

Following the initial consultation, 16 patients underwent regular rehabilitation therapy. At the most recent follow‐up (mean: 6 years; range: 6 months to 13 years), 11 patients had never achieved independent ambulation, including seven with MEB (Patients 1–3, Patients 5–8) and 4 with CMD (Patients 9, 11, 12, and 13). Notably, Patient 9 succumbed to respiratory failure secondary to pulmonary infection at 12 years and 1 month of age. In contrast, four patients (Patients 10, 14–16) exhibited only mild muscle weakness and retained ambulatory function. The remaining patient (Patient 4) was 9 months old at the last follow‐up and had not yet reached the milestone of walking age.

## DISCUSSION

DGPs represent a group of autosomal recessive disorders caused by defects in the O‐linked glycosylation of α‐DG, a key component in maintaining muscle membrane integrity and central nervous system function. The *CRPPA*‐encoded ISPD protein localizes to the endoplasmic reticulum and Golgi apparatus, where it functions as a second enzyme in the ribitol 5‐phosphate biosynthetic pathway. Through its catalytic activity, ISPD converts ribitol 5‐phosphate and CTP to CDP‐ribitol, which is subsequently utilized by ribitol 5‐phosphate transferase to modify the O‐mannose‐linked glycan chains on α‐DG. Disruption of this enzymatic cascade results in the hypoglycosylation of α‐DG, leading to the loss of its laminin‐binding capacity and compromised sarcolemmal stability.^16^, ^17^, ^18^ These disorders present with a variety of clinical symptoms, including muscular dystrophy, and often involve the central nervous system and eyes, leading to significant disability and early mortality.^19^, ^20^ In our cohort, 16 Chinese patients with *CRPPA*‐related DGPs, a condition typically presenting in early childhood, were identified, thereby broadening the clinical and genetic spectrum of this rare pediatric condition. Phenotypic variability ranged from severe MEB disease to milder congenital or limb‐girdle presentations, consistent with the wide spectrum previously reported in other populations.^10^, ^21^, ^22^, ^23^ The presence of consistent brain malformations, particularly cerebellar and brainstem dysplasia, across all patients underscores the critical role of DG glycosylation in neurodevelopment.^24^, ^25^ Notably, the predominance of severe phenotypes in our cohort highlights the substantial disease burden associated with *CRPPA* mutations.

Genetic analysis has revealed several important findings. Twenty‐one distinct pathogenic variants were identified, including five novel mutations, thereby expanding the mutational spectrum of *CRPPA*. A particularly significant discovery was the recurrent exon 6–9 deletion, present in 25.0% of our cohort and shown by haplotype analysis to represent a founder mutation in the Chinese population. A founder mutation suggests that this mutation arose from a common ancestor and was maintained in the Chinese gene pool, possibly because of genetic drift or population bottlenecks.^26^, ^27^ This frequency contrasts with the mutational patterns observed in other populations, where European cohorts are characterized by diverse point mutations with no single variant exceeding 10% frequency, and international cohorts demonstrate greater genetic heterogeneity with more dispersed mutation types.^17^, ^23^, ^28^ Notably, the exon 6–9 deletion has not been reported as a recurrent variant in non‐Asian populations, indicating its unique presence in the Chinese gene pool. This population‐specific pattern observed here warrants further investigation in larger cohorts. Although the exon 6–9 deletion was identified in 25.0% of our patients, the limited sample size precludes definitive conclusions regarding the utility for population‐based screening strategies. Nonetheless, awareness of this recurrent deletion may aid clinicians in targeted genetic testing, particularly when WGS or multiplex ligation‐dependent probe amplification is performed in Chinese pediatric patients with suspected DGP. Larger epidemiological studies are essential to determine the true population frequency of this founder mutation and to evaluate the clinical and economic justification for targeted screening. Interestingly, although previous reports have emphasized variant clustering in the catalytic domain, a substantial proportion of mutations in our series were localized in the C‐terminal non‐catalytic region, suggesting that both domains play critical roles in *CRPPA* function.^12^, ^22^

Our genotype–phenotype correlation analysis revealed both expected and unexpected patterns when compared to the broader DGP literatures. While severe truncating mutations (particularly frameshift variants like the exon 6–9 deletion) were predominantly associated with severe phenotypes, as anticipated, our compound heterozygous cohort demonstrated substantially greater phenotypic heterogeneity than typically reported in homozygous populations.^17^ The considerable phenotypic variability observed even among patients with identical variants—ranging from severe MEB to milder CMD presentations—cannot be fully explained by allelic combinations alone and likely reflects the influence of genetic modifiers or environmental variables.^10^, ^23^ These findings underscore a critical limitation in current genotype‐based prognostication and highlight the need for more sophisticated predictive models that incorporate both allelic architecture and broader genomic context.

The dystrophic histopathology and α‐DG hypoglycosylation observed in our patients' muscle biopsies are consistent with the established pathological hallmarks of DGPs.^29^, ^30^ However, the degree of IIH6 immunoreactivity loss varied among patients, which aligns with emerging evidence that residual glycosylation capacity may correlate with phenotypic severity.^31^ Importantly, the preserved merosin expression in our cohort distinguishes *CRPPA*‐related DGPs from merosin‐deficient CMD (MDC1A),^32^, ^33^ reinforcing the concept that distinct molecular mechanisms can converge on similar dystrophic pathology. This phenotypic convergence despite mechanistic diversity reflects the central role of the dystrophin‐glycoprotein complex in maintaining sarcolemmal integrity across different forms of muscular dystrophy.^34^

The striking mitochondrial abnormalities observed in our patients‐swelling, cristae disorganization, and morphological heterogeneity‐raise an important question: do these represent direct effects of *CRPPA* deficiency or secondary consequences of dystrophic processes? The evidence favors the latter interpretation. Mitochondrial dysfunction has emerged as a convergent downstream pathway across structurally distinct muscular dystrophies.^35^, ^36^, ^37^, ^38^ In Duchenne muscular dystrophy, calcium dysregulation secondary to sarcolemmal instability triggers mitochondrial stress.^39^ Similarly, in collagen VI‐related muscular dystrophies, mitochondrial dysfunction and defective autophagy have been identified as important pathological mechanisms contributing to disease progression.^35^ Mitochondrial dysfunction has been reported in various forms of muscular dystrophy as a secondary consequence of primary structural defects.^35^, ^39^ For *CRPPA*‐related DGP, we propose that α‐DG hypoglycosylation initiates a cascade: compromised sarcolemmal integrity disrupts calcium homeostasis, chronic degeneration‐regeneration cycles impose oxidative stress, and heightened metabolic demands of regenerating fibers overwhelm mitochondrial capacity.^37^, ^38^, ^40^ Critically, this may establish a self‐amplifying pathological loop—mitochondrial failure further impairs membrane repair and regenerative efficiency, accelerating disease progression. These observations carry therapeutic implications beyond glycosylation correction. If mitochondrial dysfunction amplifies phenotypic severity, interventions targeting mitochondrial quality control or calcium handling might provide synergistic benefits. Correlating mitochondrial pathology with clinical heterogeneity could identify patient subgroups most amenable to such strategies and clarify whether *CRPPA* has unrecognized mitochondrial functions that warrant further investigation.

Longitudinal follow‐up further illustrated the progressive nature of *CRPPA*‐related DGPs. Two‐thirds of patients never achieved independent ambulation, and one patient died prematurely due to respiratory complications, highlighting the severity of disease progression. Nonetheless, a subset of patients retained walking ability and presented with relatively mild phenotypes, suggesting that early rehabilitation and comprehensive care may influence long‐term outcomes. These findings underscore the importance of multidisciplinary management and early intervention strategies.

This study had several limitations that must be acknowledged. The relatively small sample size reflects the rarity of the disorder, which may restrict the generalizability of our conclusions. Furthermore, although haplotype analysis strongly suggested a founder mutation for the exon 6–9 deletion, additional population‐level studies are needed to confirm its prevalence in the broader Chinese population. Future functional studies are required to elucidate the mechanisms underlying phenotypic variability and to evaluate potential therapeutic strategies.

In conclusion, our study provides a comprehensive analysis of the clinical, genetic, and pathological features of *CRPPA*‐related DGP in a Chinese population. The identification of a founder mutation for exon 6–9 deletion has immediate implications for diagnostic practice, while the recognition of mitochondrial abnormalities expands the pathophysiological framework of the disease. Together, these findings contribute to an improved understanding of *CRPPA*‐related DGP and will inform both clinical management and future research.

## CONFLICT OF INTEREST

The authors declare no conflict of interest.

## Supporting information



Supporting Information
