# Supplementary material for: CRPPA exon 6–9 deletion as a founder mutation in Chinese patients with dystroglycanopathy
Source: Pediatr Investig. 2025 Nov 30;10(1):25–37. doi: 10.1002/ped4.70029 (PMC12921627; doi:10.1002/ped4.70029)
Supplement: Supplementary file 1 — Supporting Information [file PED4-10-25-s001.pdf]

Supplementary Material for

***CRPPA* exon 6-9 deletion as a founder mutation in Chinese patients with dystroglycanopathy**

Jihang Luo, Yidan Liu, Danyu Song, Shiqi Yang, Xiaona Fu, Lin Ge, Cuijie Wei, Liya Cui, Yanbin Fan, Huaxia Luo, Yanwei He, Jin Xu, Qiang Shen, Yuxuan Guo, Motoi Kanagawa, Tatsushi Toda, Jingmin Wang, Hong Zhang, Hui Xiong

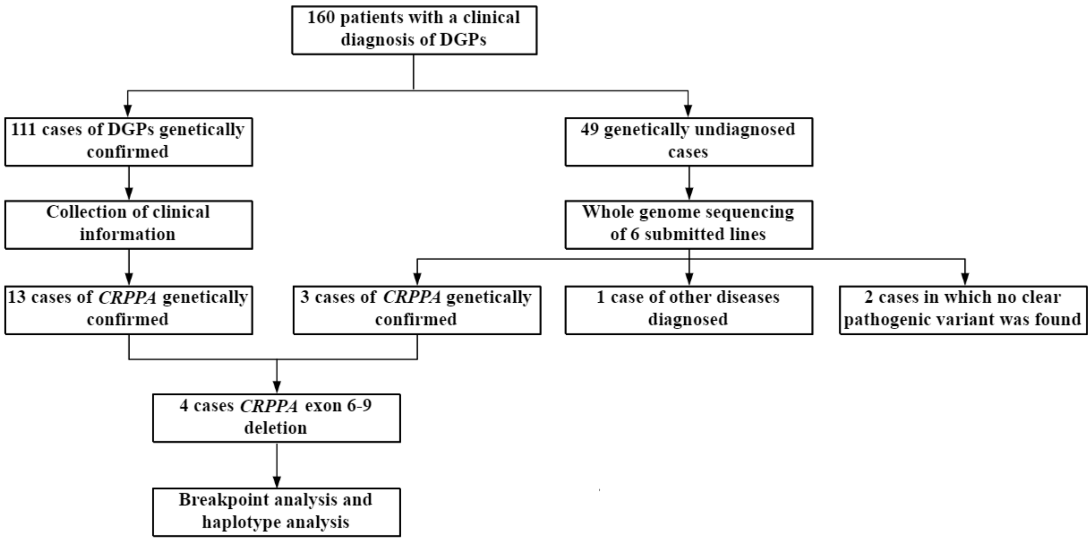

Figure S1. Workflow for diagnosis and analysis of patients with clinically proposed DGPs. Sixteen patients with *CRPPA*-related DGPs were confirmed by WES and WGS, and a breakpoint analysis and haplotype analysis were performed. DGPs, dystroglycanopathies; WES, whole-exome sequencing; WGS, whole-genome sequencing.
